# Comprehensive Orthodontic Treatment of Adult Patient with Cleft Lip and Palate

**DOI:** 10.1155/2014/795342

**Published:** 2014-12-03

**Authors:** Noemí Leiva Villagra, Miguel Muñoz Domon, Sebastian Véliz Méndez

**Affiliations:** Unit of Craniofacial Malformations, Faculty of Dentistry, University of Chile, Avenue Suecia 1033, Providencia, 7510355 Santiago de Chile, Chile

## Abstract

The aim of the paper is to present full orthodontic treatment of an operated cleft lip adult patient. *Case Report*. An 18-year-old patient consulted for severe crowded teeth. He comes from a poor family. At that time he already had four operations (velum, palate, lip, and myringotomy). Treatment included maxillary expansion, tooth extraction, and fixed orthodontic, as well as kinesiology and speech therapy treatment. A multidisciplinary approach allowed us to achieve successfully an excellent result for this patient and gave him a harmonic smile and an optimal function without orthognathic surgery. Two years after treatment, occlusion remains stable.

## 1. Introduction

Cleft lip and cleft palate are considered to be one of the most common birth defects involving craniofacial structure. Case incidence varies worldwide between 0,55 and 2,55/1000 newborns born alive (NBA) [1, 2]. In Chile, this rate is estimated to be 1/580 NBA. Unilateral cleft lip is almost eight times more frequent than bilateral and twice more frequent on the left side. Etiology is multifactorial, where both genetic and environmental factors play a part in it [3]. This anomaly not only has its aesthetic consequences but also affects different functions, depending on whether it is cleft lip or cleft palate. Complete clefts have an effect on feeding, hearing, nasal breathing, and phonation. All of these aspects are addressed as part of an integral treatment.

The current treatment protocol is based on the fact that the greater number of issues should be addressed early and decisively if possible. The most significant advances in the treatment of cleft lip and palate happen with the development of the multidisciplinary teams that approach jointly and in a coordinated manner all aspects of this complex anomaly in order to obtain good results. This allows all team members to become acquainted with the different aspects of this pathology and to coordinate the treatment more effectively. This interaction has enabled the comprehensive management of the disease with excellent results.

The objectives of the orthodontic treatment of a malocclusion on a cleft patient are the same and are considered important on any other case, achieving functional efficiency, structural equilibrium, and aesthetic harmony. In adult patients with orofacial clefts, most of the published cases involve orthodontic treatment with orthognathic surgery or even prosthetic treatment [4–6], demanding a very high economic cost for the patient.

The aim of this paper is to present the case of an 18-year-old patient, with operated unilateral cleft lip palate, treated with a multidisciplinary team approach.

## 2. Case Report

An 18-year-old male patient came to the Craniofacial Malformation Unit consulting for orthodontic treatment, with his crowded teeth as the main problem. He was born with unilateral cleft lip palate on the right side and at the time of his visit he had gone through four operations: velum (8 months old); palate (1 year old); lip (2 years old), and myringotomy (12 years old). He was the second child of two brothers and at the time of birth the mother was 30 and the father was 32 years of age. Their economic situation was meager.

At an extraoral examination, the patient presented vertically three thirds proportioned. Furthermore, there is a proportion on the lower third part of the face where the upper lip takes up the upper third and the lower lip and chin the two lower thirds. In a transversal direction, we get proportioned fifths, good lip closure, and a slightly retractable scar. In a lateral view, the nose shows good projection, with a slight hump on the dorsum. Root, dorsum, columella, and nasolabial angle were normal, as well as lip and chin projection. The lip scar is mild; however, it has affected the development of the nose. The right nostril is vertical and the left one is horizontal and narrow. The apex is conveniently not deviated as it usually occurs in these cases.

At intraoral examination, maxillary shows a surgery scar along the palate with bilateral compression of 4 m, especially at 1.5, which has no space in the upper arch. Rotated tooth 1.4; 1.3 is in high position with lack of space; verified agenesis of 2.2; 2.3 is mesially rotated and supernumerary tooth on the palate. In the jaw there is a lack of space to align 4.3 and 4.4 with a mild incisor crowding and 3.5 without any space. The occlusion on the right side presented the first molars and canine teeth in distoclusion, but over the left side we lost the occlusal plane due to crowding. Central incisors are vertical, loss of space that leaves 3.5 in infraocclusion and 3.6 in distoclusion. There is coincidence between centric relation and centric occlusion. With group function, without any anterior distoclusion guidance.

**Figure 1:**
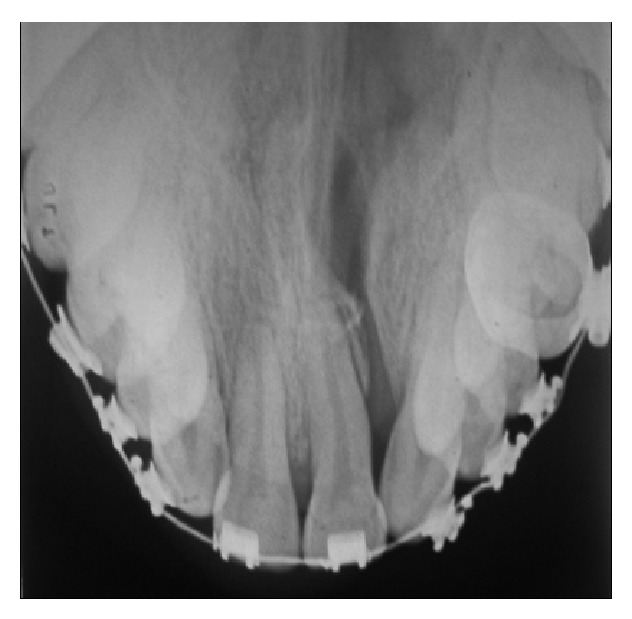
Occlusal radiograph. You can see the cleft through the palate compromising alveolar ridge and hard palate.

At orthopantomography and occlusal radiograph (Figure 1), a cleft is observed with a 1.2 agenesis as well as the presence of supernumerary located on the mesial 2.3. Third molars are observed in intraosseous evolution without any space in the arches. Cephalometric analysis showed a facial convexity of 3 mm and ANB angle of 3° gave us retruded mandible but very mild regarding the maxilla, with a component of mandibular clockwise rotation. Mild skeletal class II with severe upper maxillary transverse compression. Regarding soft tissue, there is a good ratio between middle and lower thirds of the face and good upper and lower lip projection. Study models analysis revealed a lack of maxillary space of −26 mm and a discrepancy of −10 mm.

Functional examination made by a speech therapist revealed that the problem was that he had articulating /P/; /S/; /T/; /F/; and /R/ phonemes in Spanish language and tongue lowered at rest.

Treatment goals included uncross biting with slow maxillary expansion, exodontia of 1.5, lateral incisor of 1.2, and supernumerary, aligned and leveled, with both arches keeping soft tissue healthy, maintaining good facial harmony, improved dental aesthetic with orthodontics and cosmetic rehabilitation, motor reeducation of atypical deglutition, and improvement of speech deficiency.

**Figure 2:**
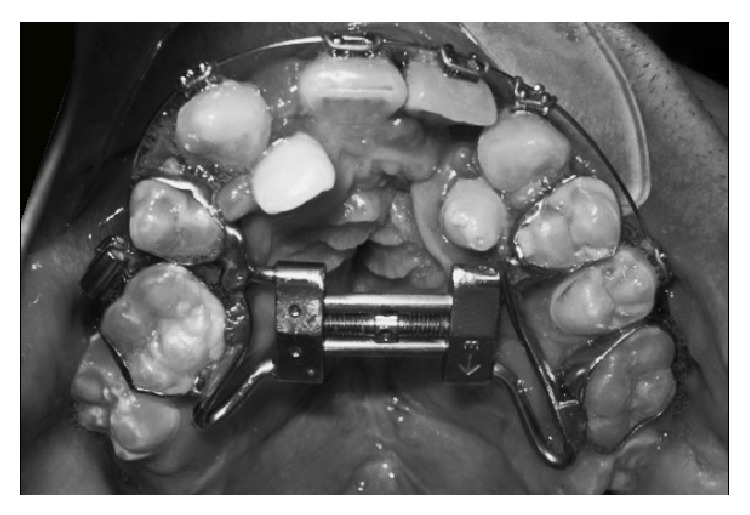
Type hyrax disjunction device. Intraoral image of disjunctor. You can observe that the exodontia of 1.5 has already been performed, but not the others yet (lateral incisor 1.2 and supernumerary teeth). Behind the device you can see the mucous scar of the cleft.

Because of the severe discrepancy, being greater on the maxilla than on the mandible, and great maxillary compression, it was necessary to do extractions of 1.5. This was done at the beginning in order to have space on the arch. A Hyrax disjunctor (Figure 2) is installed with slow maxillary expansion up to 13 mm. It is kept for 5 months and then braces are installed on the upper arch. After reevaluating the case with study models, it is decided to extract 1.2 and the supernumerary, thus achieving harmony on the upper arch, leaving both canine teeth as lateral incisors. On the mandible, it was decided to do exodontia of the first premolars and to install lower braces. Orthognathic surgery was not incorporated to treatment because the discrepancy in sagital jaw relations was small.

During treatment, the patient was in speech therapy for 10 months to solve the problem that he had articulating, regarding phonemes /P/ and /F/ specifically. Exercises were conducted with pharyngoplasty at 22 years old, whose aim was to achieve better occlusion of the velum. Kinesiological treatment was performed to perform motor reeducation of atypical deglutition.

**Figure 3:**
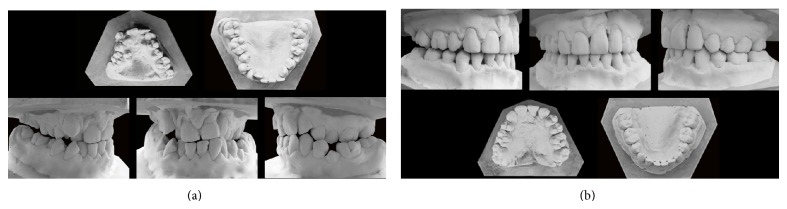
Dental cast at the beginning (a) and at the end (b) of the treatment. You can see the dental crowding in the maxilla, with the presence of 1.5 on the hard palate. Negative discrepancy was solved through extractions and slow maxillary expansion.

**Figure 4:**
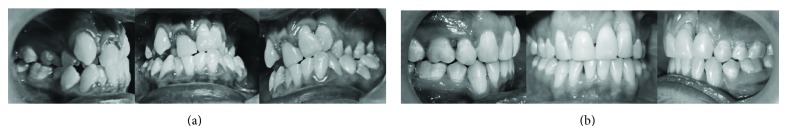
Intraoral images at the beginning (a) and at the end (b) of the treatment. Canines were remodeled as lateral incisors with composite.

After a two-year treatment with conventional fixed orthodontic, patient's braces were removed and fixed retention was installed in upper and lower arch (Figures 3 and 4).

**Figure 5:**
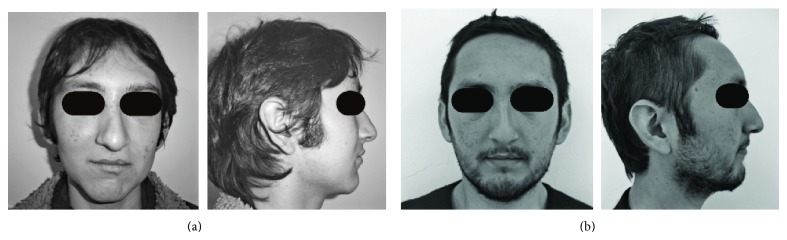
Extraoral images at the beginning (a) and at the end (b) of the treatment. No major changes were made after orthodontic treatment. The scar of the upper lip is slightly perceived.

**Figure 6:**
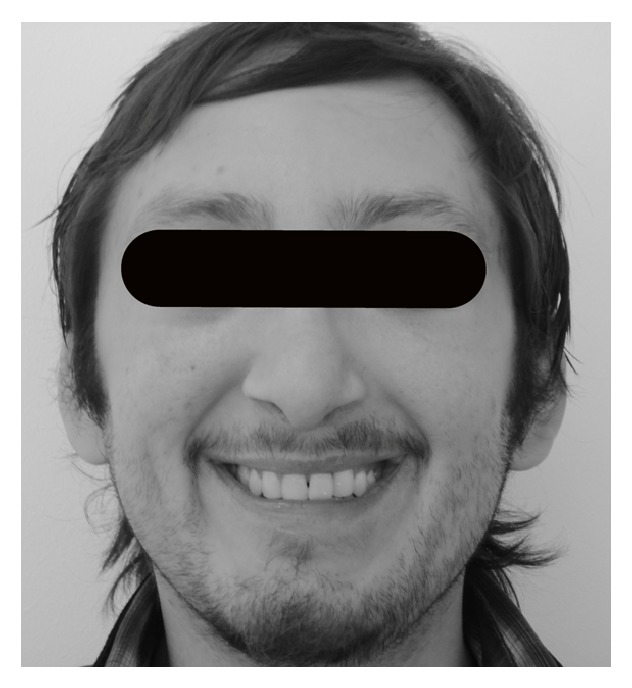
Smile picture after a two-year followup. There is a harmonic smile and the aesthetic remodeled canines look naturally as lateral incisors.

There was no major extraoral change after the treatment (Figure 5). A two-year followup shows the stability during time of the treatment. Tongue position remains correct after treatment and speech issues are better after pharyngeal surgery (Figure 6).

## 3. Discussion

Cleft lip palate represents a very common malformation, with a very wide amount of physically associated implications as speech problems, kinetics issues, and feeding troubles. But just as important are the physiological problems that this malformation carries. It is common to see problems such as low self-esteem and social interactions difficulties. And if we add to all of these economic problems and lack of access to treatment, most of these issues will remain during adulthood [7].

This patient has economic problems and recently entered an engineering school, so the possibility of any surgery was beyond his ability to pay. Most of the protocols for cleft patients involve orthognathic surgery, alveolar bone grafting, osteogenic distraction, or even palatal closure [8–10]. This carried us to think of a low cost treatment for a very severe maxillary compression.

The patient presented a nongrafted alveolar cleft and the teeth adjacent to this had in average a 3 mm recession with another 3 mm of periodontal probing before and after the periodontal treatment, but never showed mobility, pain, or sensitivity (Figures 1 and 4). In spite of this, it is important to emphasize the fact that most of the tooth adjacent to a nongrafted alveolar cleft, central incisor, and canine present a marked gingival recession and lack of bone support, mainly interproximally and labially. In this case, fixed retention may allow for better teeth stability in cases of major mobility and bone lost. There are many bone graft procedures for alveolar cleft patients, depending on each case, but in general bone graft provides a good alveolar continuity, closure of oronasal fistula, support for the nasal base, and bone support for the later teeth eruption [11, 12].

Without any doubt the most important factor in the treatment of this pathology is the multidisciplinary handling by an expert group of specialists in the matter with good interaction in decision making. The permanent concern for achieving better results is what has allowed us to reach the current situation of early handling and the primary correction of lip, nose, and gingiva with primary surgery. This radical change in the initial management has created a breakthrough in the results with a significant decrease in the side effects. To move forward the teams evaluate their treatment protocols on a regular basis so this way they can objectively guide the management of this malformation, achieving progress towards an increasingly optimal management of cleft lip and palate.
